# Percutaneous Treatment of Mitral Regurgitation After Failed Mitral Transcatheter Edge-to-Edge Repair

**DOI:** 10.3390/jcdd12120472

**Published:** 2025-11-30

**Authors:** André González-García, Julio Echarte-Morales, Manuel Barreiro-Pérez, José Antonio Baz-Alonso, Andrés Íñiguez-Romo, Rodrigo Estévez-Loureiro

**Affiliations:** 1 Department of Cardiology, University Hospital Alvaro Cunqueiro, C/Clara Campoamor 341, 36213 Vigo, Spain; andre.gonzalez.garcia@sergas.es (A.G.-G.); juliocecharte@gmail.com (J.E.-M.); manuelbarreiroperez@gmail.com (M.B.-P.); jose.antonio.baz.alonso2@sergas.es (J.A.B.-A.); andres.iniguez.romo@sergas.es (A.Í.-R.); 2 Cardiovascular Research Group, Department of Cardiology, University Hospital Alvaro Cunqueiro, Fundación Biomédica Galicia Sur, Servizo Galego de Saude, University of Vigo, 36312 Vigo, Spain

**Keywords:** mitral regurgitation, mitral valve repair, mitral valve replacement, percutaneous mitral valve repair, transcatheter mitral edge-to-edge repair, transcatheter mitral valve replacement

## Abstract

Mitral regurgitation is one of the most prevalent valvular heart diseases globally and the second most common indication for cardiac valve surgery, surpassed only by aortic stenosis. Over the past decades, open-heart mitral valve surgery has been the gold-standard intervention for this complex disorder, but in recent years, transcatheter edge-to-edge repair has emerged as a valuable option in selected clinical scenarios. However, a considerable proportion of patients develop recurrent mitral regurgitation during follow-up, leading to a significant increase in morbidity and mortality. In this context, data is limited regarding the optimal approach. This review provides an overview of the current evidence on transcatheter mitral valve intervention therapies for the management of recurrent mitral regurgitation following transcatheter edge-to-edge repair.

## 1. Introduction

Mitral regurgitation (MR) is currently the second most commonly treated heart valve disease (HVD), following aortic stenosis [1]. Its prevalence increases with age, meaning the number of cases is expected to rise as the population ages. MR is also frequently associated with other valvular diseases [2] and it can result from both anatomical and/or functional abnormalities affecting any part of the mitral valvular apparatus. Understanding the underlying mechanism of MR is crucial due to its prognostic implications and its impact on treatment strategies [3].

Although surgical mitral valve repair (SMVr) is well documented for its safety and efficacy in PMR, only a small proportion of patients with symptomatic severe SMR undergo surgery, primarily due to concerns about high surgical risk and poor outcomes [4,5]. This treatment gap has driven the development of mitral transcatheter edge-to-edge repair (M-TEER), which is now recognized as a safe and effective alternative for patients in selected clinical scenarios [6,7]. The recently published European guidelines for the management of HVD have upgraded the recommendation levels for M-TEER in both primary and secondary MR [8].

Despite being a safe, reproducible technique with a high rate of immediate procedural success, a non-negligible percentage of patients (up to 30%) experience recurrent MR during follow-up, leading to increased morbidity and mortality [9,10]. Conservative management of these patients is associated with poor outcomes, and surgical reintervention is rarely performed due to high surgical risk [11,12]. In this context, percutaneous mitral valve intervention techniques have emerged as viable alternatives for high-risk patients [13,14].

This review aims to discuss the current percutaneous therapeutic options available for managing recurrent MR after failed M-TEER.

## 2. Current Indications for Mitral Transcatheter Edge-to-Edge Repair in Mitral Regurgitation

M-TEER currently represents the standard of care for patients with SMR and serves as a less-invasive alternative for patients with PMR who are at high risk for conventional surgery. However, the mitral valve (MV) anatomy is complex, and not all MV structures are tailored for M-TEER [15]. Certain anatomical features may hinder the success of M-TEER with a MitraClip (Abbott, Santa Clara, CA, USA), including calcified leaflets, smaller valve areas, and short posterior leaflets, which can limit the ability to achieve optimal outcomes [16]. New devices such as the PASCAL system (Edwards Lifesciences, Irving, CA, USA) were designed to overcome some of these limitations. A recent metanalysis demonstrated comparable efficacy and safety between the PASCAL and MitraClip systems at short- and mid-term assessments [17]. Other devices are entering the field with great results, such as the case of the DragonFly device (Valgen MedTech, Hangzhou, China), which shares features with the MitraClip and PASCAL. The DragonFly DMR trial, a prospective and ongoing registry, has showed encouraging results [18].

That said, with the increasing number of M-TEER procedures and subsequent patient follow-ups, the recurrence of MR after M-TEER (Table 1) and its clinical impact will likely become a common scenario that must be adequately managed, including identifying predictors of recurrent MR.

### Clinical Impact of Recurrent Mitral Regurgitation After Mitral Transcatheter Edge-to-Edge Repair

Since its initial adoption, the use of M-TEER has expanded dramatically, with cases increasing nearly 10-fold within the first five years, and this growth trend appears to be ongoing. Based on this data, a good selection of candidates and an optimal procedural result are extremely important (Table 2) [26,27,28]. The clinical setting, procedural strategy, and mechanisms of failure differ substantially between primary (degenerative) and secondary (functional) MR. In primary MR, leaflet pathology such as prolapse or flail is the main driver of regurgitation, and recurrent MR after TEER usually results from leaflet-related issues, including loss of leaflet insertion or leaflet tear. Conversely, in secondary MR, regurgitation reflects ventricular or annular remodeling, and recurrence is often functional, without structural device failure.

In a large multicenter analysis, flail leaflet and residual MR ≥ 2+ were independent predictors of recurrence in primary MR, whereas left atrial enlargement and residual MR ≥ 2+ predicted recurrence in secondary MR [10] (Table 3). These findings underscore the importance of evaluating and reporting outcomes according to MR etiology. While recurrent MR is associated with adverse prognosis in both groups, the pathophysiological mechanisms and therapeutic strategies—such as the feasibility of redo M-TEER versus need for surgical or transcatheter replacement—differ considerably. Therefore, whenever possible, outcomes and management strategies after TEER should be interpreted in the context of the underlying MR etiology.

This affirmation is supported by real-world data from the STS/ACC TVT Registry published by Sorajja et al. [20] because one of the variables associated with poor outcomes was a worse post-procedural MR (MR ≥ 3+) which occurred in 7% of the patients. Compared to patients with residual MR ≤ 2+, those with residual MR ≥ 3+ had significantly higher in-hospital mortality (10.2% vs. 2.1%) and an increased 1-year mortality rate (48.9% vs. 23.9%). For MR grade ≤ 1+, grade 2+, and grade ≥ 3+, the cumulative incidences of death at 1 year were 21.7%, 29.2%, and 48.9%, respectively [20]. Regarding the composite endpoint of all-cause death HFH, these respective 1-year incidences were 35.7%, 39.2%, and 54.4%. In addition, repeat M-TEER was performed in 6.2% of the cohort and patients with an unsuccessful post-implant result required more frequent subsequent procedures. MVS was performed in 6.1% of these patients within one year, compared to 1.7% in those with residual MR ≤ 2+, while redo M-TEER was necessary in 17.9% of cases, versus 5.1% in patients with residual MR ≤ 2+. Finally, poorer procedural results were associated with higher rates of acute complications, such as single-leaflet device attachment (7.0% vs. 1.0%) [20].

In this line, Adamo et al. [29] demonstrated an association between an optimal procedural result and favorable outcomes in patients with SMR who underwent M-TEER. This large Italian multicenter registry showed that patients with an optimal procedural result (MR ≤ 1+), compared with those with an acceptable result (MR 2+), had better prognosis with lower rates of all-cause and cardiac deaths (25.7% vs. 40%, *p*  < 0.001 and 16.3% vs. 24.8%, *p*  = 0.003, respectively) and HFH (24% vs. 30%; *p*  = 0.035) at 2-year follow-up [29]. This has been consistently demonstrated in other real-world registries with different devices. At 1-year follow-up in the EXPAND registry [30], all-cause mortality was 17.7% among patients with SMR, and the combined endpoint of all-cause mortality or first HF hospitalization occurred in 34% of patients and was significantly less frequent in patients with MR ≤ 1+ compared with MR ≥ 2+ (29.7% vs. 69.6%, respectively) [30].

A total of 809 patients with SMR included in the EuroSMR registry were evaluated by Higuchi et al. [31]. Achieving an optimal MR reduction (MR ≤ 1+) was associated with improved survival, especially if the progress in HF is not too advanced compared with an acceptable MR reduction (MR 2+). Post-procedural MR was also significantly associated with survival at 2 years. In comparison with MR ≤ 1+, MR 2+ demonstrated a strong trend with mortality (unadjusted HR: 1.34; *p* = 0.056), while MR ≥ 3+ was significantly associated with increased mortality (unadjusted HR: 1.69; *p* = 0.032) [31]. Finally, from the OCEAN-Mitral registry, 2150 patients who underwent M-TEER were analyzed and stratified in three different groups based on residual MR severity at discharge: MR 0+/1+, 2+, and 3+/4+. At 1-year follow-up, mortality and HFH rates in the entire cohort were 12.3% and 15.0%, respectively. Imaging and clinical improvements were sustained, with 94.1% of patients having MR ≤ 2+ and 95.0% in NYHA class ≤II. However, higher residual MR at discharge was associated with worse outcomes: MR 2+ and MR ≥ 3+ were linked to increased death or HF hospitalization compared with MR ≤ 1+ (adjusted HR: 1.59 and 1.73, respectively). In addition, NYHA class III/IV at 1 year was more frequent in MR ≥ 3+ (20%) vs. MR ≤ 1+ (4.6%) and MR 2+ (6.4%) and more patients in MR ≤ 1+ (57.8%) achieved NYHA class I compared to MR 2+ (48.3%) [32].

Collectively, data from multiple large registries consistently highlight the prognostic impact of residual mitral regurgitation after M-TEER. Patients achieving an optimal procedural result (MR ≤ 1+) experienced markedly lower rates of mortality and heart failure hospitalization compared with those with residual MR ≥ 2+. Overall, these findings demonstrate that the extent of MR reduction translates directly into improved survival, fewer heart failure events, and better symptomatic outcomes, emphasizing the importance of achieving optimal procedural results and careful patient selection in M-TEER.

## 3. Management of Patients Who Experience Failed Mitral Transcatheter Edge-to-Edge Repair

**Figure 1:**
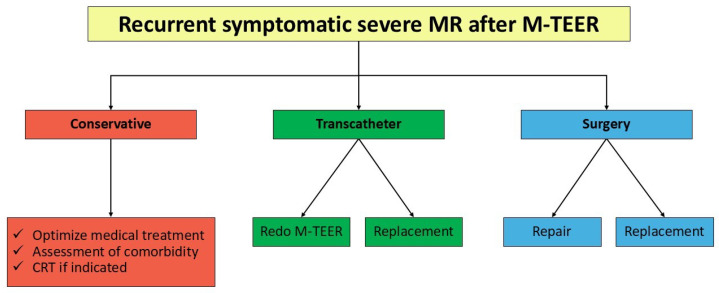
Treatment alternatives for recurrent mitral regurgitation after transcatheter edge-to-edge repair. CRT: cardiac resynchronization therapy; MR: mitral regurgitation; M-TEER: mitral transcatheter edge-to-edge repair.

Persistent and recurrent severe MR occurs in approximately 4–15% of patients after M-TEER [14,36,37,38,39]. Medical, surgical, and percutaneous interventions have all been suggested as potential solutions for this issue (Figure 1).

### 3.1. Conservative, Surgical, or Percutaneous Intervention: Which Treatment Strategy to Choose?

**Figure 2:**
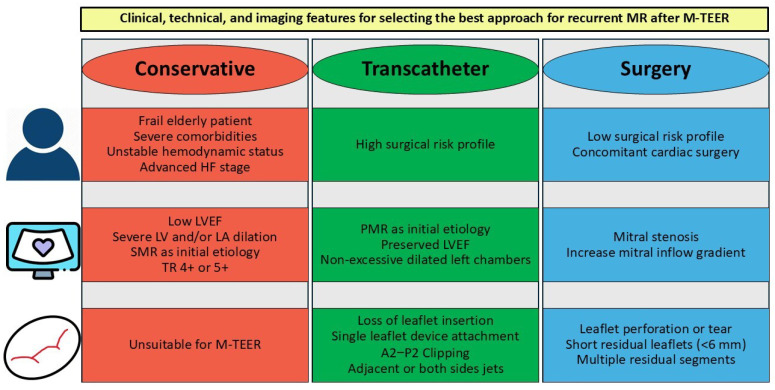
Key clinical, technical, and imaging features for selecting the most appropriate therapeutic approach. Recommendations for the management of recurrent severe MR following M-TEER vary depending on patient characteristics and anatomical findings. Conservative management is reserved for patients with limited life expectancy, where the risks of intervention outweigh the potential benefits. Transcatheter treatment, particularly redo M-TEER, is strongly recommended in patients without leaflet damage, without multiple regurgitant jets (e.g., a single jet adjacent to the previous device or bilateral jets amenable to a “sandwich” technique), and without extensive adverse ventricular remodeling. Surgical intervention—most often involving valve replacement—is indicated for patients with low surgical risk and features such as mitral stenosis or increased mitral inflow gradient (>5 mm Hg), leaflet perforation or tear, short residual leaflets, and/or multiple jets. LA: left atrial; LV: left ventricle; LVEF: left ventricular ejection fraction; MR: mitral regurgitation; M-TEER: mitral transcatheter edge-to-edge repair; PMR: primary mitral regurgitation; SMR: secondary mitral regurgitation; TR: tricuspid regurgitation.

The current situation regarding the best approach to treat patients with significant recurrent MR after M-TEER is still not clear (Figure 2).

**Figure 3:**
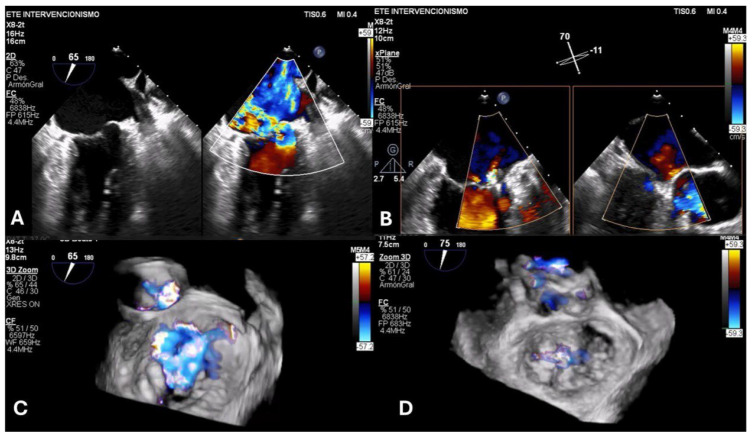
Redo M-TEER intervention. Transesophageal echocardiography interventional study (Courtesy of Li CP, MD). (**A**): Dual 2D and color Doppler imaging: Severe PMR before the index M-TEER procedure, secondary to P1 prolapse. (**B**): X-Plane color Doppler imaging: Optimal procedural result after M-TEER between A1 and P1. (**C**): 3D imaging: Severe recurrent MR one year after M−TEER. (**D**): 3D imaging: Final result after redo M-TEER with an additional device placed from A1−P1 to the nearby A2−P2 segment.

**Figure 4:**
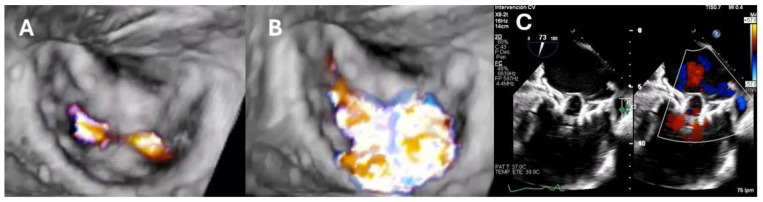
Transcatheter mitral valve replacement after M-TEER. Transesophageal echocardiography interventional study: (**A**): 3D imaging of the mitral valve apparatus in diastole. Clip device placed in the A2–P2 position. (**B**): 3D imaging of the mitral valve apparatus in systole. Mitral regurgitation grade 4+ consisting of two main jets (lateral and central). (**C**): 2D and color Doppler imaging after clip removal and mitral valve replacement performed with Tendyne (Abbott Vascular, Plymouth, MN, USA).

Patients with prior M-TEER can be stratified according to the presence or absence of SLDA and by the timing of presentation (early or delayed). In cases of SLDA, a thorough assessment of the mechanism and location of the residual MR jet(s) is essential. In fact, new or previously untreated valve pathology may be identified and treated with a repeat TEER procedure. Redo M-TEER (Figure 3) should generally be avoided in cases where leaflet integrity is already compromised, such as in the presence of perforation or tear, as it may further deteriorate the leaflet tissue. Additionally, short leaflets (<6 mm) present a challenge for adequate leaflet grasping. Broad or extensive leaflet pathology also reduces the likelihood of procedural success with M-TEER. Finally, significant mitral stenosis (MS) with a mitral valve area ≤ 2.0 cm^2^ and an elevated mitral inflow gradient (≥5 mmHg) often renders patients unsuitable for M-TEER [40] (Figure 4). Therefore, the use of advanced imaging techniques is essential to achieve a comprehensive evaluation and plan a potential redo M-TEER intervention.

#### 3.1.1. Current Evidence on the Most Appropriate Therapeutic Approach After Failed M-TEER

Ikenaga et al. analyzed 41 patients who underwent repeat MV intervention (surgical or percutaneous) after M-TEER. In patients with PMR before M-TEER, the main etiologies of recurrent MR were worsening leaflet prolapse at the clip site (50%) and leaflet tear at the clip site (29%). Meanwhile, in patients with SMR before M-TEER, LV and/or LA dilation with worsening tenting were the most common causes of recurrent MR [35]. In another small cohort of 37 patients (mean age 75 ± 8.9 years, 46% male) with significant residual MR (MR ≥ 3+) after failed M-TEER, treated between January 2013 and January 2018 and who were admitted for HF, medical treatment alone resulted in poor 1-year prognosis with a 12.5% survival rate vs. 47.6% in the MVS group vs. 50% in the redo M-TEER group [41]. Therefore, repeat M-TEER and MVS may offer improved survival, particularly in patients without prohibitive surgical risk. However, the selection of treatment modality should be individualized, taking into account the underlying valve pathology and patient comorbidities.

In a recent unicentric, retrospective observational study, El Shaer et al. presented 142 symptomatic patients with persistent or recurrent severe MR after M-TEER who were ineligible for redo M-TEER. Among them, 44 individuals (31.0%) were treated with surgical mitral valve replacement (SMVR) in high volume centers and the rest of the cohort were managed conservatively. Patients who underwent surgery were younger than those treated medically (74.1 ± 8.9 vs. 78.6 ± 10.5 years, *p* = 0.01) but had a similar STS score (9.0 ± 4.7% vs. 7.9 ± 4.9% in the surgical and medical therapy groups, respectively; *p* = 0.22). Concomitant surgery was performed in all cases and included atrial septal defect closure, atrial fibrillation ablation, tricuspid valve repair, or coronary artery bypass grafting (CABG). In the SMVR group, an operative mortality rate of 4.5% was achieved (observed/expected operative mortality ratio = 0.55) and postoperative complications were uncommon. After risk adjustment, the SMVR group had a three-fold-lower all-cause mortality rate compared to the medical therapy group [12].

Giordano et al. [42] collected data from a large Italian multicenter registry and analyzed a total of 2238 patients who underwent M-TEER. Of them, 96.9% had no prior mitral intervention, 1.3% had undergone previous MitraClip implantation, and 1.8% had a history of SMVR. Patients with prior interventions had a higher prevalence of NYHA functional class IV symptoms (13.8% vs. 8.7%, *p* = 0.001). Of note, STS score was lower in patients with previous SMVR compared to those undergoing M-TEER. Additionally, the prior M-TEER group showed lower LVEF and a higher rate of implantable cardioverter-defibrillator (ICD) use, indicating a higher-risk clinical profile. In terms of MR etiology, the M-TEER group had a greater proportion of SMR, while PMR predominated in the SMVR cohort. Although not statistically significant, the group with prior M-TEER had lower procedural success rates and a higher incidence of residual MR ≥ 2+ compared to both native valve and SMVR groups. Nevertheless, despite these trends, the authors showed that M-TEER was still largely successful in this population, achieving comparable rates of HFH and mortality at a median follow-up of 14 months, so repeat M-TEER (whether after prior TEER or surgical repair) had high procedural safety and mid-term efficacy, with comparable outcomes to those in patients undergoing first-time intervention. This supports M-TEER as a viable therapeutic option after previous MV repair failures [42].

Data extracted from the Society of Thoracic Surgeons Adult Cardiac Surgery Database (STS-ACSD) between July 2014 and June 2020 indicated that SMVr is infrequently (less than 5%) achieved after failed M-TEER, which may have implications for treatment choice in lower-risk and younger patients [43]. Late SMVr is technically challenging because the devices are designed to induce a proliferative response, generating fibrotic tissue that secures the clip. This process quickly leads to significant sclerosis, as well as thickening and distortion of the leaflets and subvalvular apparatus [41]. Chikwe et al. [43] analyzed a total of 463 patients: 38.2% had degenerative disease, the median age was 76 years, and the median STS score was 6.5%. SMVR was performed in 95.2% of the cohort (91.8% bioprostheses). Concomitant surgery in addition to MVS was performed in 237 patients (51.2%). The subsequent operative mortality was relatively high at 10.6%; however, it was significantly lower (2.6%) in the quintile of hospitals with the most experience in these procedures. Notably, for patients with underlying degenerative disease undergoing elective surgery, the mortality rate was only 2.8%. In multivariate analyses, urgent status, nondegenerative or unknown etiology, preoperative creatine >2.0 mg/dL, and age >80 years were independent predictors of the primary outcome [43].

From Medicare beneficiary data, Kaneko et al. identified 548 patients (4.8% of the total cohort) who required reintervention after M-TEER with a median time interval of 4.5 months from the index TEER procedure. Among them, 294 (53.7%) patients underwent repeat M-TEER and 254 (46.3%) underwent MVS. Patients who underwent MVS were younger, but had a similar comorbidity burden compared with the redo M-TEER group. There were no differences in 30-day mortality or 30-day readmission between groups. However, MVS was associated with nearly a five-fold-higher 30-day morbidity (defined as the composite outcome of pneumonia, transfusion requirement, stroke, acute kidney injury, and cardiac arrest) compared with redo M-TEER. Analyzing the whole cohort, requirement for reintervention was an independent risk factor for long-term mortality [44]. So, these findings underscore the importance of optimizing the success of the initial procedure to minimize the need for reintervention in this high-risk profile population.

Another recent single-center study, which retrospectively analyzed 52 real-world patients (median age 81 years), demonstrated the feasibility and safety of redo M-TEER for recurrent significant MR (50% SMR). All redo procedures were completed successfully with device deployment, without any intraprocedural complications or the need for conversion to surgery. Additionally, the study showed improved symptomatic status and a reduction in regurgitation severity at both 1 month and 1 year following the procedure among patients with available follow-up data (*n* = 24). The 1-year composite rate of all-cause death or HFH was 26.9%, closely aligning with the rate observed in patients undergoing M-TEER for the first time and a worse outcome was associated with SMR etiology and independently predicted by the presence of significant TR and non-A2P2 clipping [45].

Mangieri et al. [46] evaluated the incidence, management, and outcome of patients who experienced M-TEER failure secondary to loss of leaflet insertion (LLI), SLDA, or embolization after MitraClip implantation between 2009 and 2020. The global incidence of this phenomenon was 3.5% and had a similar distribution between MR etiologies. LLI occurred in 31.9% of the cases, SLDA in 67.3%, and embolization only once (0.8%). Moreover, the majority of failures were detected before discharge (60.5%). In total, 80 (54.4%) subjects underwent a redo procedure, either M-TEER (34.7%) or surgically (24.5%), including four cases of surgical conversion of the index procedure and seven cases of bailout surgery after unsuccessful redo M-TEER, so the rate of effective percutaneous redo was 74.5%. The rest of the patients (45.6%) were conservatively managed. The median follow-up was 162 days, and significant MR occurred in 43.9% of the patients, with a death rate of 29.3%. The redo M-TEER strategy demonstrates a trend toward a reduced risk of death (21.6% rate during the follow-up) compared with bailout surgery (28.0% death rate) and conservative management (35.2% death rate). Independent predictors of mortality included postprocedural acute kidney injury, advanced age, and moderate-to-severe TR [46].

New techniques are under development, with one of the most promising being TMVR as seen in several case reports [47,48,49,50]. A small case series of only five patients with severe MR or stenosis despite M-TEER showed the feasibility of device detachment from the anterior leaflet with electrosurgical laceration and subsequent TMVR with the Tendyne (Abbott Vascular) bioprosthesis [51].

The ELASTA-Clip technique (Electrosurgical Laceration and Stabilization of Clip devices) is a percutaneous method (transapical access) developed to manage failed TEER when TMVR is planned. It involves creating a single functional mitral orifice by releasing the anterior leaflet from the TEER device while preserving its attachment to the posterior leaflet. This approach avoids the technical complexity and risks associated with full device explantation and facilitates proper seating of the TMVR prosthesis, provided the residual clip does not interfere with valve deployment. The technique uses a coordinated system of catheters and electrified guidewires known as the “flying V” to lacerate the mitral leaflets. Under general anesthesia and TEE guidance, the failed device is first snared and stabilized. Electrosurgical energy is then applied to cut the posterior and anterior leaflets sequentially, freeing the device from both leaflets while maintaining control of the clip. ELASTA-Clip expands the therapeutic options for high-risk patients with failed TEER and may serve as an alternative to surgical explantation in selected cases.

Recently, Samim et al. [52] published their multicentric experience in 22 patients (mean age 77.8 years, mean STS score 7.2%) with failed M-TEER who underwent ELASTA-Clip followed by compassionate-use or commercial transapical TMVR using the Tendyne system. Technical success was achieved in 21 patients (95.4%). Optimal MR reduction was achieved in 89.5% of the cases and at 30 days, all patients were alive. Three patients (15.0%) were rehospitalized for HF, with uncontrolled AF in two cases. Additionally, one patient underwent a reintervention for valve tensioning. Some weaknesses of this procedure include the high rates of paravalvular leak progression and stroke at 30 days, both of which had a 15.0% incidence [52].

In a recent case report from Elison et al. [49], a new variant from the ELASTA-Clip technique was described with the first in-human successful percutaneous excision of a failed MitraClip device followed by TMVR. This procedure was justified because in certain TMVR devices like the M3 valve (Edwards Lifesciences) the residual clip may obstruct valve implantation, necessitating complete explantation. The procedure was performed under general anesthesia with transesophageal echocardiographic (TEE) guidance and hemodynamic support via a prophylactic intra-aortic balloon pump. Two large-bore sheaths were placed in the right femoral vein: one to accommodate the retrieval system, which used a nitinol snare within a retrieval basket (ŌNŌ, ŌNŌCOR Vascular) to grasp and stabilize the MitraClip, and the other to support the laceration system, which utilized the “flying V” configuration of electrified guidewires directed toward the medial and lateral mitral orifices. After securing the clip, sequential electrosurgical laceration was performed—first cutting the posterior mitral leaflet, then repositioning the system to lacerate the anterior leaflet—effectively freeing the failed TEER device in preparation for transcatheter mitral valve replacement TMVR [49].

Another promising therapy is the AMX Clip Removal System (AMX Technologies). It is an innovative, investigational device designed to address this growing need for treating failed transcatheter edge-to-edge repair (TEER) in mitral and tricuspid valves [53]. Using a catheter-based electrosurgical approach, the system enables precise, minimally invasive detachment and removal of implanted TEER clips via a transseptal pathway, reducing procedural risk and recovery time. By clearing the valve area of obstructive clip devices, it creates a viable path for subsequent transcatheter valve replacement, significantly expanding treatment options for high-risk patients. The system embodies a “repair-first” strategy aligned with modern valve therapy, preserving future intervention possibilities while offering an immediate solution for recurrent or residual regurgitation after TEER failure. The AMX Clip Removal System features proprietary radiofrequency technology with both single and multiple electrode configurations, allowing for precise and controlled detachment of TEER clips. This flexibility enhances procedural safety and effectiveness by tailoring energy delivery to the specific anatomy and clinical scenario [53].

Finally, learnings from the patients who underwent MVS after failed M-TEER included in the CUTTING-EDGE international registry showed that the initial etiology of MR impacted the prognosis after surgery [54]. A total of 330 patients were included, of which 155 patients (47%) had PMR and 175 patients (53%) had SMR. The mean age at the time of the index TEER procedure was 73.8 ± 10.1 years, 42.7% were female, and the median STS score was 4.0%. SMR patients had a significantly lower LVEF pre-TEER and post-TEER. Also, SMR patients had a shorter posterior leaflet length at the location of device and more frequently received the original MitraClip^®^, with fewer MitraClip^®^ NTs. Additionally, SMR individuals had a larger mitral valve area (5.2 ± 2.1 cm^2^ vs. 4.3 ± 1.8 cm^2^; *p* < 0.039 pre-TEER) and a greater comorbidity burden compared with PMR patients: higher EuroSCORE II (5.1% vs. 3.8%; *p* = 0.004), coronary artery disease (58.3% vs. 42.6%; *p* = 0.006), diabetes mellitus (38.9% vs. 23.9%; *p* = 0.004), AF (64.0% vs. 52.3%; *p* = 0.034), chronic kidney disease (62.3% vs. 41.3%; *p* <0.001), and prior pacemaker/defibrillator implantation (38.9% vs. 22.6%; *p* = 0.002). The median time interval from the index TEER procedure to MV surgery was 3.5 months overall, with no significant differences between MR groups (*p* = 0.74), and the overall median follow-up was 17.8 months from the index TEER procedure and 9.1 months after MVS, with no differences between groups. More aborted M-TEER cases were observed in SMR patients (25.7% vs. 16.2%). The surgical indications were recurrent MR (33.6%), residual MR (28.8%), LLI (25.2%), partial detachment (21.5%), MS (14.5%), and clip embolization (2.1%). There were no significant differences between the two groups, except that MS was a more frequent reason for MVS among patients with SMR. They also underwent fewer MV repairs, and had a worse prognosis compared to those with PMR (1-year mortality rate 38.3% vs. 23.2%; *p* = 0.019). Moreover, TR severity at the time of the index M-TEER procedure was an independent predictor of 1-year mortality in patients with SMR. Finally, chronic kidney disease, preoperative MR severity, and emergent surgery were identified as independent risk factors for 1-year mortality in the PMR cohort. In the SMR group, male sex, cirrhosis, pre-TEER TR severity, and cardiopulmonary bypass time were independent predictors of 1-year mortality [54].

In conclusion, both redo M-TEER and also MVS (especially replacement surgery) are feasible and safe alternatives in patients without an excessively increased perioperative risk. Whenever leaflet insertion is not compromised, repeat M-TEER is a reasonable redo concept; in the remaining cases, surgery should be offered to the patient. Data on TMVR in this context is limited, although initial results highlight safety concerns that need to be addressed [52], although the development of novel technologies that would enable the safe removal of TEER devices creates a promising opportunity to improve clinical decision-making and to establish TMVR as a feasible and effective therapeutic alternative.

#### 3.1.2. Early Versus Delayed Failure

The timing of failure strongly influences both prognosis and therapeutic opportunities. *Early failure* (intra-procedural or within 30 days) is typically related to technical or anatomical issues, including inadequate leaflet grasping, SLDA, leaflet perforation, or device embolization. In these cases, bail-out strategies such as re-grasping, additional clip implantation, or urgent surgery may be required. By contrast, delayed failure (beyond 30–90 days) is more often associated with progressive leaflet pathology, ventricular remodeling, or annular dilation. These patients usually present with recurrent symptoms and provide a therapeutic window for more comprehensive evaluation and planned intervention. Delayed failure allows consideration of redo M-TEER, surgical repair or replacement, or, in selected high-risk patients, emerging transcatheter valve replacement options.

#### 3.1.3. Patient Selection and Decision-Making Pathways

Appropriate patient selection is central to optimizing outcomes and ensuring rational use of healthcare resources. A redo M-TEER may be considered in patients with preserved leaflet integrity, single or anatomically suitable regurgitant jets, adequate leaflet length, and without significant transmitral gradients. Conversely, patients with broad leaflet pathology, short or torn leaflets, or elevated mitral inflow gradients (>5 mmHg) are unlikely to benefit from repeat clipping and should be referred for surgical intervention if surgical risk is acceptable. In low- to intermediate-risk patients, surgery could be a valuable option, whereas in prohibitive-risk patients, transcatheter replacement and clip-removal strategies may be considered in highly specialized centers. Importantly, comorbidities, frailty, and expected survival must always be factored into decision-making, reserving conservative management for patients where intervention is futile.

## 4. Conclusions

As M-TEER becomes more widely used and patients are followed over longer periods, the number of repeat procedures is expected to rise because conservative management is related with poor outcomes, and most patients needing an additional procedure can still achieve a good prognosis. However, a standardized approach for the optimal method of reintervention has yet to be established.

## Figures and Tables

**Table 1:** Procedural results of M-TEER at discharge and at 1-year follow-up.

Study	Authors	MR Etiology (N, %)	Procedural Success ^1^	MR ≥ 3+ at 1 Year
COAPT	Stone et al. [19]	SMR (614, 100%)	95%	5.2%
STS/TVT Registry	Sorajja et al. [20]	PMR (2536, 85.9%) SMR (254, 8.6%) Mixed (262, 8.9%)	92%	N/A
EXPAND	Kar et al. [21]	PMR or mixed (526, 50.5%) SMR (515, 49.5%)	95.8%	2.5%
EXPAND G4	von Bardeleben et al. [22]	PMR (424, 43.0%) SMR (554, 48.3%)	97.9%	1.6%
CLASP IID	Zahr et al. [23]	PMR (294, 100%)	97.4%	4.9%
DRAGONFLY-DMR	Wang et al. [18]	PMR (120, 100%)	99.2%	8%
MATTERHORN	Baldus et al. [24]	SMR (102, 100%)	N/A	3.9%
RESHAPE	Anker et al. [25]	SMR (250,100%)	92.2%	9.1%

**^1^**: acute procedural success is considered as a MR ≤ 2+ at discharge. MR: mitral regurgitation; M-TEER: mitral transcatheter edge-to-edge repair; N/A: not available; PMR: primary mitral regurgitation; SMR: secondary mitral regurgitation.

**Table 2:** Clinical impact of recurrent mitral regurgitation after M-TEER.

Study	MR Reduction	MR Reintervention and/or Outcomes
Sugiura et al. [10]	MR ≤ 2+ at 1 year, 90.2% vs. MR ≥ 3+, 9.8%	HFH or NYHA III/IV: 37.8% vs. 54.1% (*p* = 0.018) Repeat MV intervention: 2.2% vs. 9.8% (*p* = 0.005) Mortality ^1^: 16.1% vs. 42.3% (*p* = 0.08)
Sorajja et al. [20]	MR ≤ 2+ post-procedural, 93.0% vs. MR ≥ 3+, 7.0%	MVS: 1.7% vs. 6.1% (*p* <0.001) Redo M-TEER: 5.1% vs. 17.9% (*p* <0.001) Mortality ^2^: 23.9% vs. 48.9% (*p* <0.001)
Adamo et al. [29]	Post-procedural MR 0/1+, 67% vs. MR 2+, 33%	HFH ^3^: 24.0% vs. 30.0% (*p* = 0.035) CV death ^3^: 16.3% vs. 24.8% (*p* = 0.003) Mortality ^3^: 25.7% vs. 40% (*p* < 0.001)
Orban et al. [30]	MR ≤ 1+ at discharge, 92.0% vs. MR ≥ 2+, 8.0%	Mortality or HFH: 29.7% vs. 69.6% (*p* < 0.001) HFH: 22.2% vs. 62.2% (*p* < 0.001) Mortality ^2^: 15.8% vs. 29.6% (*p* < 0.001)
Higuchi et al. [31]	Post-procedural MR 0/1+, 67% vs. MR 2+ 26% vs. MR, ≥3+ 7%	Mortality ^3^: 29% vs. 36% vs. 41%
Kubo et al. [32]	MR severity at discharge, MR ≤ 1+ 75.8% vs. MR 2+ 20.5% vs. MR ≥ 3+ 3.7%	HFH ^2^: 13.4% vs. 19.8% vs. 21.0% (*p* = 0.001) MV reintervention ^2^: 1.6% vs. 2.4% vs. 19.0% (*p* < 0.001) Mortality ^2^: 10.3% vs. 18.9% vs. 16.9% (*p* < 0.001)

^1^ At 3-year follow-up. ^2^ At 1-year follow-up. ^3^ At 2-year follow-up. HFH: heart failure hospitalization; MR: mitral regurgitation; MV: mitral valve; M-TEER: mitral transcatheter edge-to-edge repair; MVS: mitral valve surgery; NYHA: New York Heart Association.

**Table 3:** Main predictors of recurrent MR after mitral transcatheter edge-to-edge repair.

MR Etiology	Predictors
All	Impaired renal function (GFR < 60 mL/min/1.73 m^2^) [33] High pulmonary artery pressure (PASP > 50 mm Hg) [33] Restricted leaflet motion (Tethering angle of posterior leaflet > 45°) [33] Mitral stenosis [33] Residual MR ≥ 2+ upon discharge [10] AP diameter of LV inflow orifice ≥ 3.5 cm [34] Low volume centers [29]
Primary	Flail leaflet as primary etiology [35] Leaflet prolapse or tear at the clip site [35]
Secondary	MR 4+ at baseline [29] Larger LA volume [35] Larger LVEDV (>70 mm) [35] Lower LVEF [29]

AP: anterior–posterior; BMI: body mass index; GFR: glomerular filtration rate; LA: left atrial; LV: left ventricle; LVEDV: left ventricular end-diastolic volume; LVEF: left ventricular ejection fraction; MR: mitral regurgitation; PASP: pulmonary artery systolic pressure.

## Data Availability

No new data were created or analyzed in this study.

## Abbreviations

The following abbreviations are used in this manuscript:

AF	Atrial fibrillation
AP	Anterior–posterior
BMI	Body mass index
CABG	Coronary artery bypass grafting
CRT	Cardiac resynchronization therapy
EuroSMR	European Registry of Transcatheter Repair for Secondary Mitral Regurgitation
GDMT	Guideline-directed medical therapy
GFR	Glomerular filtration rate
HF	Heart failure
HFH	Heart failure hospitalization
HVD	Heart valve disease
ICD	Implantable cardioverter-defibrillator
LA	Left atrial
LLI	Loss of leaflet insertion
LV	Left ventricle
LVEDV	Left ventricular end-diastolic volume
LVEF	Left ventricular ejection fraction
MR	Mitral regurgitation
MS	Mitral stenosis
M-TEER	Mitral transcatheter edge-to-edge repair
MV	Mitral valve
MVS	Mitral valve surgery
NYHA	New York Heart Association
PMR	Primary mitral regurgitation
PASP	Pulmonary artery systolic pressure
SLDA	Single leaflet device attachment
SMR	Secondary mitral regurgitation
SMVR	Surgical mitral valve replacement
SMVr	Surgical mitral valve repair
STS-ACSD	Society of Thoracic Surgeons Adult Cardiac Surgery Database
STS/ACC TVT	Society of Thoracic Surgery/American College of Cardiology Transcatheter Valve Therapy
TEER	Transcatheter edge-to-edge repair
TR	Tricuspid regurgitation
